# Gold Coast Criteria in ALS Diagnosis: A Real-World Experience

**DOI:** 10.3390/brainsci14111055

**Published:** 2024-10-25

**Authors:** Lucia Ferullo, Barbara Risi, Filomena Caria, Emanuele Olivieri, Loris Poli, Stefano Gazzina, Ugo Leggio, Enrica Bertella, Giorgia Giovanelli, Beatrice Labella, Alessandro Padovani, Massimiliano Filosto

**Affiliations:** 1 Department of Clinical and Experimental Sciences, University of Brescia, 25121 Brescia, Italy; l.ferullo@unibs.it (L.F.); e.olivieri002@unibs.it (E.O.); beatrice.labella93@gmail.com (B.L.); alessandro.padovani@unibs.it (A.P.); 2 Unit of Neurology, ASST Spedali Civili, 25123 Brescia, Italy; loris.poli@asst-spedalicivili.it (L.P.); stefano.gazzina@asst-spedalicivili.it (S.G.); ugo.leggio@asst-spedalicivili.it (U.L.); 3 NeMO-Brescia Clinical Center for Neuromuscular Diseases, 25064 Brescia, Italy; barbara.risi@centrocliniconemo.it (B.R.); filomena.caria@centrocliniconemo.it (F.C.); enrica.bertella@centrocliniconemo.it (E.B.); giorgia.giovanelli@centrocliniconemo.it (G.G.)

**Keywords:** amyotrophic lateral sclerosis, motor neuron disease, Gold Coast criteria, revised El Escorial criteria, Awaji criteria

## Abstract

**Background:** Revised El Escorial (rEEC) and Awaji criteria are currently used for diagnosing and categorizing amyotrophic lateral sclerosis (ALS). However, they are complex; their sensitivity is still not optimal for research purposes, and they present high inter-rater variability in clinical practice. To address these points, in 2019, a new set of diagnostic criteria was proposed, namely the Gold Coast criteria (GCC), characterized by a dichotomous diagnostic categorization, i.e., ALS or not ALS. **Methods:** In order to investigate the sensitivity, specificity, and clinical usefulness of GCC in a practical clinical setting, we retrospectively evaluated 131 patients diagnosed with ALS and 104 control subjects. ALSFRS-R score, electrophysiological tests, neuroradiological investigations, and CSF analysis were obtained. rEEC, Awaji, and GCC were applied at the first and last evaluations. **Results:** The sensitivity of GCC (93.1%; 96.1%) was greater than rEEC (71.8%; 87%) and Awaji criteria (77.8%; 89.3%) both at the first visit and last follow-up. The GCC’s specificity (28.8%) is lower than that of the other two criteria (rEEC 45.2%; Awaji 43.3%). **Conclusions:** Our study suggests that in a real-world setting, the GCC are more sensitive and have substantially lower risk of false negative diagnoses than rEEC and Awaji criteria. Although rEEC had the highest specificity, they may delay the diagnosis. Systematically using the GCC could help to achieve an earlier diagnosis and quickly refer patients to the correct management. The low specificity of GCC is likely to not significantly impact patient recruitment in clinical trials; therefore, its use might allow a faster and earlier enrollment.

## 1. Introduction

Amyotrophic lateral sclerosis (ALS) is a fatal neurodegenerative disease and the most common form of motor neuronal disease (MND) [1]. Incidence increases with age and is highest between 60 and 79, although variations in age of onset are widely described in clinical practice [2,3,4,5].

Diagnosing ALS can be challenging because there is no single test that can definitively confirm the disease. Although diagnostic tools may usefully support the diagnostic suspicion, the diagnosis of ALS is still based on the clinical evaluation, i.e., the presence of signs of upper motor neuron (UMN) and lower motor neuron (LMN) damage [6].

Over time, there has been an evolution in the criteria used for diagnosing ALS [1].

For a considerable period, the revised El Escorial (rEEC) World Federation of Neurology criteria served as the clinical benchmark [7,8]. They emphasized the presence of both upper and lower motor neuron signs, as well as the progression of symptoms over time, and established four levels of diagnostic certainty: definite, probable, possible, and suspected. To enhance diagnostic sensitivity, the rEEC introduced a category of ‘laboratory-supported probable ALS’, allowing electromyographic (EMG) data to complement clinical observations [8].

Subsequently, the Awaji criteria were introduced to improve diagnosis, especially in early ALS [9]. They integrated EMG data with clinical findings, considering the neurophysiological features of LMN dysfunction as equivalent to LMN clinical signs. Furthermore, the inclusion of fasciculations as an LMN sign and fasciculation potentials as additional LMN signs led to erasing the clinically probable laboratory-supported ALS category [9,10].

Although several studies have shown that the Awaji criteria are more sensitive than the rEEC, their sensitivity remains suboptimal for research purposes, and both sets of criteria are not easy to use in the routine clinical setting due to their complexity and significant inter-rater variability [10,11,12,13].

In view of these limitations, a consensus meeting was convened in the Gold Coast, Australia, which proposed a new set of simplified diagnostic criteria for ALS [14]. The novel criteria aim to render the diagnostic categories dichotomous, i.e., ALS or not.

The Gold Coast criteria’s (GCC) requirements for ALS diagnosis are (1) a documented history or repeated clinical assessments that demonstrate progressive motor impairment after a period of normal motor function; (2) the presence of both upper and lower (clinical or EMG) motor neuron signs in at least one body region (or UMN and LMN dysfunction in the same body region if only one region is affected), or LMN dysfunction in at least two body regions; (3) thorough investigations must be conducted to rule out any other potential disease process (Table 1).

Even though the GCC are simpler and aim to facilitate early diagnosis of ALS, their reliability compared to previous ones is still an ongoing matter of debate [15].

In this study, we evaluated the sensitivity, specificity, and clinical usefulness of the GCC compared to the rEEC and Awaji criteria in a real-world clinical setting to assess their reliability.

## 2. Patients and Methods

Patients and controls were recruited at the Unit of Neurology, ASST Spedali Civili (Brescia, Italy), and NeMO-Brescia Clinical Center for Neuromuscular Diseases (Brescia, Italy) between June 2012 and May 2023.

For each subject, we collected (1) demographic information and clinical features, including the region of onset of the symptoms and duration in months between first and last visits; (2) the presence or absence of UMN signs, such as hyperreflexia, spasticity and pseudobulbar aspects, and LMN signs, such as focal weakness, wasting, fasciculations and hyporeflexia; (3) data from investigations including brain and spine MRI, blood tests, cerebrospinal fluid tests, and genetic tests; (4) electromyography (EMG) findings encompassing spontaneous activities (fibrillation potentials, positive sharp waves, and fasciculation potentials) and qualitative or quantitative Motor Unit Potential (MUP) analysis; (5) diagnostic classification according to GCC, Awaji and rEEC.

Clinical staging was conducted during the initial and final follow-up assessments by the Amyotrophic Lateral Sclerosis Functional Rating Scale—Revised (ALSFRS-R) [16]. The duration of the disease (measured in months) from the initial visit to the last follow-up was documented.

The diagnosis of ALS was determined in accordance with good clinical practice which required a consistent disease progression with established ALS clinical criteria, as already described [17]. Additionally, the exclusion of potential mimic disorders was performed by clinical evaluation, neurophysiological tests, laboratory tests, and neuroimaging assessment [17].

Patients who have been diagnosed with other conditions or who have not experienced progression for at least 12 months were classified as non-ALS. For each set of criteria, sensitivity and specificity were calculated.

### Statistical Analysis

Analyses were performed for the total population of ALS patients and the control group. The primary endpoint was to estimate the diagnostic utility (sensitivity and specificity) of the GCC in distinguishing ALS from ALS-mimicking diseases compared with the Awaji and rEEC, as defined by the proportion of patients categorized as definite, probable, or possible ALS. For detecting an increase in sensitivity from 70% to 80% or more, a confidence level of 95% was used. McNemar’s test was applied to test for differences in sensitivity and specificity between the criteria. *p* values < 0.05 were considered statistically significant. A secondary analysis was also performed to estimate the risk of false positive or false negative diagnoses of ALS by calculating the positive predictive value (PPV) and negative predictive value (NPV) for each criterion. Statistical analyses were performed using IBM SPSS statistics version 25.

## 3. Results

### 3.1. Clinical Features

**Figure 1:**
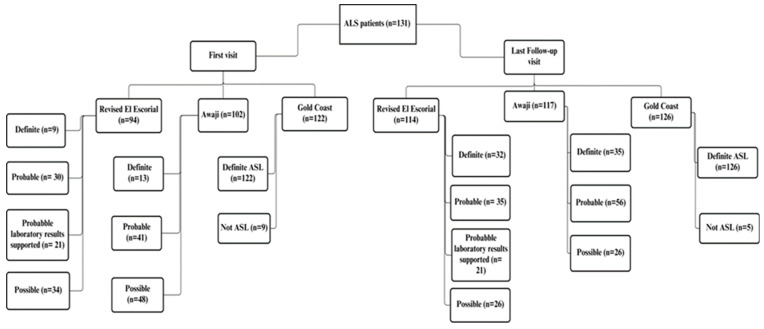
Flow diagram of ALS patients diagnosed based on rEEC, Awaji criteria, and GCC.

We retrospectively evaluated 235 patients. Out of these, 131 patients were diagnosed with ALS, and 104 control subjects had no diagnosis of ALS at the end of the diagnostic process. A flow diagram of ALS patients diagnosed based on rEEC, Awaji criteria, and GCC is reported in Figure 1. Table 2 displays the demographic data.

Our cohort consists of 141 males and 94 females with a mean age of 62.5 ± 12.2 years. After extensive clinical, laboratory, neurophysiological, and radiological examination, including follow-up, 131 patients were diagnosed with ALS (74 men and 57 women, mean age at onset = 63.6 ± 10.3 years) and 104 patients had no final ALS diagnosis (67 men and 37 women, mean age at onset = 61.1 ± 14.2 years). 

ALS diagnosis was based on clinical and neurophysiological findings and serial clinical longitudinal follow-up evaluation for at least six months with evidence of disease progression.

At the last follow-up visit, the mean duration of the disease in ALS patients from the initial visit was 16.5 ± 14.9 months. Of these 131 patients, 89 (68%) exhibited limb onset and 42 (32%) exhibited bulbar onset. In the control group, all patients experienced the onset of symptoms in the limbs (Table 2).

### 3.2. Diagnostic Criteria Comparison

In our population, GCC demonstrated higher sensitivity (93.1%; 95% confidence interval [95%CI] = 88–96.6) compared to rEEC (71.7%; 95%CI = 63.7–79) and Awaji criteria (77.8%; 95%CI = 70.3–84.4) both at the first visit and last follow-up with statistically significant differences (*p* < 0.001). Although the specificities of the rEEC (45.2%; 95%CI = 35.8–54.8) and Awaji criteria were almost the same (43.2%; 95%CI = 34–52.9), the GCC’s specificity decreased sharply (28.8%; 95%CI = 20.7–38) with a statistically significant difference (*p* < 0.001) (Table 3).

Also, considering the two subgroups of ALS patients with bulbar or limb onset, the GCC confirmed greater sensitivity at the first and last follow-up visits both for patients with bulbar onset (90.4–95.2%) and patients with limb onset (94.3–96.7%) compared to both rEEC (bulbar onset: 78.5–88%; limb-onset: 68.5–86.5%) and Awaji Criteria (bulbar onset: 73.8–85.7%; limb-onset: 80.8–91%) (Table 4). The specificity remained consistent with the findings observed in the overall study cohort, as non-ALS disease patients were exclusively limb-onset cases.

To assess the risk of false negatives and false positives, we estimated the positive predictive value (PPV) and the negative predictive value (NPV) for each diagnostic criterion. Although GCC’s PPV (62.2%) is nearly the same as the previous criteria (rEEC 62.2%; Awaji criteria 63.3%), the GCC showed a significantly higher NPV (77%) than rEEC (56%) and Awaji criteria (60.8%) (Table 3).

## 4. Discussion

One of the main challenges in diagnosing ALS has been detecting a good balance between sensitivity and diagnosis accuracy to prevent false positives. While very detailed, the rEEC are overly strict, insufficiently sensitive, leading to diagnostic delay, and unfavorable for individuals who could not be included in clinical trials or who were only able to do so relatively late [7,8,18]. Actually, they have often been considered as one of the reasons for the failure of clinical trials with drugs that demonstrated promising results in animal models but were ineffective when administered to humans in a late stage of disease [18,19].

The diagnosis sensitivity has been marginally raised but has not significantly improved by the Awaji criteria and the revised version, which includes the previous “possible ALS” category [12,15,19,20,21,22,23].

The goal of the GCC was to improve early diagnosis and streamline the diagnostic procedure.

Recent research has demonstrated that the GCC are clinically feasible and can raise diagnostic sensitivity, especially because of considering progressive muscular atrophy (PMA) as an ALS feature [24,25,26,27]. Specificity seems not to be significantly lowered, and rare false positives included patients with cervical spondylosis and Inclusion Body Myositis [18,19,20,21,22,23,24,25].

Our study confirms that the GCC are more sensitive than both the rEEC and Awaji criteria in our population, at initial as well as at last evaluations. Conversely, the GCC were less specific than the rEEC and Awaji criteria. The GCC’s sensitivity remained consistent across the subgroups defined by the site of onset (bulbar vs. limb onset). In bulbar-onset patients, no significant differences among the three set of criteria (*p* > 0.05) were observed (Table 4). This is as expected because previous criteria were also well suited for bulbar-onset cases. Differently, in patient with limb-onset disease, the GCC are more effective at the initial stages compared to both the rEEC and Awaji criteria (*p* < 0.001). A statistically significant difference was also observed between the GCC and rEEc (*p* = 0.035) in the advanced phases of disease, but not between the GCC and Awaji criteria (*p* > 0.05) (Table 5).

In summary, the GCC allow the diagnosis of ALS in the early stages of the disease by including patients who would have been excluded by the previous criteria, especially in the limb-onset phenotype. As the disease progresses, the clinical presentation of the patient becomes more evident and the diagnostic efficacy of the three criteria tends to converge.

Even if the GCC’s positive predictive value is similar to previous criteria, they have a significantly higher negative predictive value than the rEEC and Awaji criteria, demonstrating a substantially lower risk of false negative diagnoses compared to previous criteria.

A major limitation of the rEEC and Awaji criteria is the complexity of multiple diagnostic categories. Many patients do not follow the expected progression within these categories and some individuals with a pure lower motor neuron (LMN) phenotype pass away without meeting the criteria, while post-mortem analyses reveal evidence of corticospinal tract pathology [28,29,30,31]. 

The simplification of the GCC, especially the fact that LMN dysfunction in at least two regions of the body is considered a diagnostic element, is crucial in clinical practice for obtaining early diagnosis and referring the patient to the appropriate management and therapy. It also facilitates clearer communication of the diagnosis because the previous wording (definite, probable, possible, suspected) could confuse and complicate the already complex diagnostic communication process of ALS [32].

## 5. Conclusions

Finally, the present study confirms previous findings that the GCC exhibit greater sensitivity than both the rEEC and Awaji criteria, particularly in patients with limb-onset ALS, and highlights a substantially lower risk of false negative diagnoses.

The simpler GCC could be used to recruit patients for clinical trials at an early stage of the disease, potentially replacing the complex diagnostic category models used in the Awaji and rEEC criteria.

In addition, the GCC represent a significant simplification in clinical practice that could streamline the complex process of communicating ALS diagnoses and facilitate the referral of patients for appropriate medical care.

## Figures and Tables

**Table 1:** ALS diagnostic criteria.

Criteria	Revised El Escorial Criteria (rEEC)	Awaji Criteria	Gold Coast Criteria (GCC)
**Purpose**	Diagnosis and classification of ALS	Diagnosis and classification of ALS	Diagnosis and classification of ALS
**Date Established**	Revised in 2000 (earlier versions from 1998)	Revised in 2008	Revised in 2019
**Diagnostic Categories**	**Definite ALS:** Clinica evidence of upper and lower motor neuron involvement in three regions.	**Definite ALS:** Clinical and/or electrophysiological evidence of upper and lower motor neuron involvement in at least two regions.	**Definite ALS:** Clinical evidence of upper and lower motor neuron involvement in one region or lower motor neuron involvement in at least two regions.
	**Probable ALS:** Clinical evidence of upper and lower motor neuron involvement in two regions.	**Probable ALS:** Clinical and/or electrophysiological evidence of upper and lower motor neuron involvement in one or two regions.	NA
	**Probable lab. supp:** Clinical evidence of upper and lower motor neuron involvement in one region or upper motor neuron involvement alone with EMG lower motor neuron involvement in two regions.	NA	NA
	**Possible ALS:** Clinical evidence of upper and lower motor neuron involvement in one region or upper motor neuron involvement in two more regions or lower motor neuron involvement rostral to upper motor neuron.	**Possible ALS:** Clinical evidence of upper and lower motor neuron involvement in one region or upper motor neuron involvement in two or more regions or lower motor neuron involvement rostral to upper motor neuron.	NA
**Electrophysiological criteria**	Requires specific findings (e.g., electromyography) to support the diagnosis.	Emphasizes Electrophysiological findings. (Evidence of denervation and reinnervation).	Less emphasis on specific electrophysiological findings; clinical presentation is prioritized.
**Genetic Testing**	Not required but may support diagnosis if applicable.	Genetic testing is encouraged, especially for familial history of ALS.	Genetic testing is encouraged, particularly for familial history of ALS and atypical presentations.
**Application**	Primarily used for research and clinical diagnosis.	Widely used for clinical diagnosis, reflecting an update from previous criteria.	Used for clinical diagnosis and research, with updated guidelines reflecting current practices.

**Table 2:** Characteristics of the study population.

	ALS	Not ALS
**n**	131	104
Male/Female	74/57	67/37
Mean onset age (years)	63.6 (±10.3)	61.1 (±14.2)
Δ First visit/last follow-up visit (months)	16.5 (±14.9)	26 (±27.2)
Mean ALSFRS-R at first visit/last follow-up visit (points)	41.78 (±4.8)/27 (±11.8)	N.A.
Bulbar onset/Limb onset	42/89	0/104

**Table 3:** Diagnostic accuracy of ALS criteria.

Total Population	rEEC % (95%CI)	Awaji % (95%CI)	Gold Coast % (95%CI)	*p* Values
Sensitivity	71.8 (63.7–79)	77.8 (70.3–84.4)	93.1 (88–96.6)	<0.001
Specificity	45.2 (35.8–54.8)	43.3 (34–52.9)	28.8 (20.7–38)	<0.001
Positive predictive value	62.3 (54.4–69.7)	63.4 (55.7–70.5)	62.2 (55.3–68.8)	N.A
Negative predictive value	56 (45.3–66.3)	60.8 (49.5–71.4)	77 (62.2–88.2)	N.A

**Table 4:** Sensitivities in ALS patients: bulbar-onset vs. limb-onset symptoms at initial evaluation.

ALS Patients	rEEC %	Awaji %	Gold Coast %	*p* Values
Bulbar-onset sensitivity	78.5	73.8	90.4	>0.05
Limb-onset sensitivity	68.5	80.8	94.3	<0.001

**Table 5:** Sensitivities in ALS patients: bulbar-onset vs. limb-onset symptoms at last evaluation.

ALS Patients	rEEC %	Awaji %	Gold Coast %	*p* Values
Bulbar-onset sensitivity	88	85.7	95.2	>0.05
Limb-onset sensitivity	86.5	91	96.7	GGC vs. rEEC = 0.035GCC vs. Awaji > 0.05

## Data Availability

The original contributions presented in the study are included in the article; further inquiries can be directed to the corresponding author.
